# Efficacy and safety of Sri Lankan traditional medicine regimen for knee osteoarthritis: study protocol for an open-label, active comparator, randomized controlled trial

**DOI:** 10.1186/s13063-022-06903-8

**Published:** 2022-11-22

**Authors:** Himalee De Silva, Pathirage Kamal Perera, Saroj Jayasinghe, Shreenika De Silva Weliange

**Affiliations:** 1 Institute of Indigenous Medicine, University of Colombo, Colombo, Sri Lanka; 2 Department of Clinical Medicine, Faculty of Medicine, University of Colombo, Colombo, Sri Lanka; 3 Department of Community Medicine, Faculty of Medicine, University of Colombo, Colombo, Sri Lanka

## Abstract

**Background:**

Knee osteoarthritis (KOA) is the most common form of arthritis, causing disability and impaired quality of life especially in the elderly. Sri Lankan traditional medicine (STM) is widely used to treat OA, but no clinical trial evaluated on STM regimens for KOA to discuss their safety and efficacy in the treatment. The aim of this study is to compare the efficacy and safety of STM regimen for KOA in comparison to recommended conventional pain management therapy over a period of 8 weeks on relieving the condition.

**Study design:**

This is a clinical trial following a protocol-driven open-labeled randomized controlled study enrolling patients with KOA that will be conducted as a single-center trial in the National Ayurveda Teaching Hospital, Sri Lanka. Rasnadvigunabhagasaya herbal decoction (RDBD) and an herbal pill Yoaraja Guggulu were selected as the rescue medication for treating joint disorders. The two Ayurvedic dosage forms will be tested against the non-steroidal anti-inflammatory drugs tab paracetamol and tab ibuprofen as the rescue medication for their safety and efficacy. As test products for external application, oil with an herbal fomentation—Kumburuetaperumkayam Pottani (KAP)—and paste—Sandivadam Lepaya (SVL)—were selected. External applications will be tested against the diclofenac sodium gel and hot water fomentation. KOA patients will be allocated randomly into two arms, and the medications will be given orally for 60 days and externally for 30 days. The primary endpoint is the change in the score on the WOMAC after 08 weeks. WOMAC and KOOS will be recorded and compared between the two arms prior to visiting 1, at the end of 15 days and end of 30 days, and end of the 45 days and end of the second month and 3 months of follow-up. KOOS and WOMAC subscales, a pain disability index, a visual analog scale for pain and sleep quality, and a quality-of-life index are used as secondary outcome measurements.

**Discussion:**

This clinical trial will be able to provide evidence-based scientific data on Sri Lankan traditional medicine regimens in the management of KOA. This trial is expected to develop capacity to scientifically evaluate various STM that are claimed to have efficacy in treatment of various disease conditions.

**Trial registration:**

ISRCTN58050062.

## Background

Osteoarthritis (OA) has become a burden globally for the healthcare sector due to its high prevalence and incidence. Among that, knee osteoarthritis (KOA) is the most common form of arthritis causing disability and impaired quality of life especially in elderly [1]. The WHO report on priority diseases and reasons for inclusion 2018 provides an in-depth review of the various diseases or conditions including osteoarthritis in its chapter 6 which has been selected and grouped according to the nature of the pharmaceutical gap(s) associated with it [2]. The Osteoarthritis Research Society International (OARSI) also identified OA as a serious disease, and one of their major concerns are treatments for osteoarthritis [3].

Prevalence of knee pain has been reported as 25 to 56% and appears to vary according to geographical location. Prevalence of knee pain in the USA has increased by 65% between 1983 and 2005; in Germany, citizens aged 40 years and older 30.9% reported knee pain; and in Swedish adults aged 58–84 years, the prevalence of frequent knee pain was 25.1%. China reported the prevalence of knee pain of 44% in men and 56% in women aged 59 years and above [4].

In India, osteoarthritis is the second most common rheumatologic problem and prevalence of 22% to 39% [5]. OA accounts for 24% of all years lived with disability (YLD) and has been ranked as the 10th leading contributor to global YLDs. The economic burden of arthritis on patients and society is high in every country that it has been estimated and job-related indirect costs due to loss of productivity have been estimated to cost from $3.4 to $13.2 billion per year. Recent guidelines have addressed the many treatments that aim to relieve symptoms, in particular pain and improved function, and not provide a cure for OA. The treatments that are available for the management of OA have adverse effects that are not significant. There are numerous drug treatments for osteoarthritis; however, their efficacy and adverse effect profiles often limit their use. Currently, pharmacological, non-pharmacological, and surgical measures are considered in the treatment of OA with aim of reducing pain and improving the function of the knee joint, and currently, there is no known cure and no interventions available to stop the progression or manage the symptoms with an acceptable benefit to harm profile [6].

A Sri Lankan traditional medicine (STM) called *Desheeya Chikitsa* is probably the oldest form of treatment used in the country and use of STM in health maintenance and in disease prevention and treatment, particularly for chronic diseases like KOA. By reviewing most diseases and disease conditions related to OA, *Sandhi Vadam*, *Janu Gada*, and *Sandhigata Vata* are frequently used in classification of diseases stated in STM under the disease category of *Vata Roiga* [8, 9]. For this condition, there are many treatment regimens including decoctions, pills, pastes, and fermentation procedures, recorded which would be efficacious for long-term management [7–21]. Despite the importance of the OA issue, this topic has not yet been addressed in any great depth in the field of STM. In most of the clinical trials evaluated on herbal preparations as single interventions, STM regimens are widely used to treat OA, but no clinical trial evaluated on STM regimens for KOA to discuss their safety and efficacy in the treatment of KOA [22].

Individual STM treatment regimens for *Sandivadam* included a specific treatment type which often require the individual to take an active part in their own treatment at home with general and specific STM life style modification measures and nutritional advices. Less-invasive non-hospitalized procedures and low cost but efficacious methods, unnecessary to take leave from their current occupation, and ingredients used to prepared drugs were well established with research findings and freely available in Sri Lankan settings. Hence, this study considered the common STM regimen standards of practice; decoctions and pills are therapies by far the most used medicines internally. *Rasnadvigunabhagasaya* decoction which includes 26 ingredients is an herbal decoction indicated effective in reducing pain in *Sandhigata Vata*, and it is advised to take *Yogaraja Guggulu* with this decoction used as the internal medicines, and the form of fomentation (*Thevili Pottani with Oil application*) contained 10 ingredients of herbal ball and dip in five types of oil mixture (*Pasthel*): *Kumburuetaperumkayam Pottani* (KAP) and paste (*Paththu/Lepa*): *Sandivadam Lepaya* (SVL) consisting of 15 ingredients which were selected for external use as local application in this study. No scientific studies have been published that have evaluated the efficacy and safety of the abovementioned Sri Lankan traditional treatment regimen for knee OA. Therefore, this study’s main aim is to evaluate the efficacy and safety of the aforesaid Sri Lankan traditional medicine regimen in comparison to the non-steroidal anti-inflammatory drugs (NSAIDS) tab paracetamol and tablet ibuprofen internally and external application of diclofenac sodium gel and hot water fomentation used in an allopathic system in patients with symptoms of knee OA.

## Method

### Study design

This is a 2-month clinical trial following a protocol-driven open-labeled randomized controlled study that will be conducted at the National Ayurveda Teaching Hospital in Borella, Colombo, Sri Lanka, as a single-center trial. RDBD and Yogaraja Guggulu (internally) and KAP and SVL (externally) used in Sri Lankan traditional medicine as a regimen for knee OA will be the test products. The efficacy and safety of this regimen will be tested against the non-steroidal anti-inflammatory drugs (NSAIDS) tab paracetamol and tab ibuprofen internally and external application of diclofenac sodium gel and hot water fomentation. Patients with symptoms of knee OA will be allocated randomly into two arms after a 1 week run-in period, and the medications are given orally for 60 days and externally for 30 days. This study protocol was developed as required by the Standard Protocol Items: Recommendations for Interventional Trials (SPIRIT) (Additional file 1).

Ethics approval has been obtained from the ethics review committee, Institute of Indigenous Medicine (ERCIIM), University of Colombo, Sri Lanka (ERC/20/105). The trial was registered in the ISRCTN registry (trial number ISRCTN58050062) (Additional file 2).

In recent times, there has been a paradigm shift towards patient-centered care and active involvement of patients and public in research design and active participation throughout the research process. Therefore, in this trial, designing process patient involvement is acknowledged.

### Study setting

The study will be conducted in the National Ayurveda Teaching Hospital, Borella, Colombo, Sri Lanka, and Institute of Indigenous Medicine (IIM), University of Colombo, Rajagiriya, Sri Lanka. The study subjects will be recruited from the patients with symptoms of knee OA who visit to the outpatient department (OPD) of the National Ayurveda Teaching Hospital and those who come responding to a newspaper advertisement on trial requirement.

### Participants

Patients will be selected from those who are seeking treatment for knee OA. Participation in this research project is voluntary. Recruitment is done by screening for eligibility criteria (inclusion and exclusion criteria). Eligible subjects will be randomly assigned to the Sri Lankan Traditional medicine regimen and the conventional treatment regimen group.

### Inclusion criteria and exclusion criteria

#### Inclusion criteria

Diagnosis of KOA will be based on history, clinical examination findings, and classical radiological findings (ACR functional class I, II, or III) and fulfilling the American College of Rheumatology (ACR) classification criteria except that the lower age limit was reduced to 40 years, and radiographic evidence of OA will be based on the ranking score of the Kellgren-Lawrence radiographic system, and pain severity will be based on VAS.

Three American College of Rheumatology (ACR) classification criteria for knee will be used. They are including:

- The ACR clinical classification criteria of knee OA
- The ACR clinical/radiographic classification criteria of knee OA
- The ACR clinical/laboratory classification criteria of knee OA
- The ACR clinical classification criteria for knee OA

In this criterion, the presence of knee pain along with at least three of the following six items can classify the knee OA in the following patients:

- Age > 50 years old
- Morning stiffness < 30 min
- Crepitus on knee motion
- Bony tenderness
- Bony enlargement
- No palpable warmth

#### ACR clinical/radiographic classification criteria

The presence of knee pain with at least one of the following three items along with osteophyte in knee X-ray can classify the knee OA in the following patients:

- Age > 50 years old
- Morning stiffness < 30 min
- Crepitus on knee motion

#### ACR clinical/laboratory classification criteria

The presence of knee pain along with at least 5 of the following 9 items can classify the knee OA in the following patients:

- Age > 50 years old
- Morning stiffness < 30 min
- Crepitus on knee motion
- Bony tenderness
- Bony enlargement
- No palpable warmth
- RF < 1 40
- Synovial fluid compatible with OA

#### Pain visual analog score

A five-point Likert scale version with five response levels for each item, representing different degrees of intensity: none (0), mild (1), moderate (2), severe (3), and extreme (4). The maximum score is 10 points for pain; higher scores indicate more or worse symptoms, maximal limitations, and poor health. The patients will have reported a mean pain intensity in one or both knees while performing a weight bearing activity (e.g., walking, standing, climbing staircase) of ≥ 40 mm on a 100-mm visual analog scale over the 7 days before baseline assessment and provide written informed consent.

#### Radiographic evidence of OA

This will be assessed on the ranking score of the Kellgren-Lawrence radiographic system (0–4), and 1–3 grades will be considered.

#### Exclusion criteria

Subjects who have non-degenerative joint diseases or other joint diseases such as rheumatoid arthritis, psoriatic arthritis, gonococcal arthritis, and hemoarthritis

#### Subjects with severe disabling arthritis and/or the patient is bedridden

Those that had a history of intra-articular knee injection within the month preceding the study

Those with evidence of severe unstable renal, hepatic, diabetic, hemopoietic, cancer, hypertensive, and cardiac disorder and mentally affected as revealed by history and/or investigation.

Concurrent anti-coagulant/antiplatelet drugs, corticosteroids, or narcotic pain killers use; history of epilepsy or bleeding disorders; gastric ulcers; renal or hepatic disease; uncontrolled hypertension; or body mass index (BMI) > 45 kg/m^2^.

A washout period of 7 days was required for NSAIDs users. Discontinuing the use of common CAM therapies for arthritis (e.g., glucosamine, chondroitin sulfate, bromelain, DMSO (*dimethyl sulfoxide*), acupuncture, and Ayurveda medicine) was required for 7 days prior to enrollment.

#### Dropout criteria

Participants whom for whatever reason discontinues their participation in the study will be registered and reported in our results as dropouts. If an available reason for dropout will be reported, all data related to the participant’s participation will be deleted unless the participant consents to the continual use of existing data.

#### Sample size

Sample size was calculated based on the primary outcome measurement of WOMAC Index 10-point improvement of score from baseline after 8 weeks between both parallel groups of the clinical trial. The sample size calculated was 63 with a 80% power (pooled standard deviation = 20, two-sided alpha = 0.05) using the formula *N* = 2δ^2^(*Z*_crit_ + *Z*_pow_)^2^/*D*^2^, where *N* is the sample size for each group, *δ*^2^ is the variance of either group (assumed to be equal for both groups), and *D* is the minimal detectable difference between the two means. The *z*_crit_ and *z*_pow_ are the standard normal deviations at a level of significance and at 1-*β* power, respectively. With an expected dropout rate of 10%, the minimum sample size was calculated as 70 for one arm.

#### Recruitment of patients

People who are interested in participating in this clinical study will be provided with a detailed Information sheet supplemented by verbal explanation of the study procedures. If the participants agree with the information given, recruitment of patients is the same process as described in the protocol for the trial “Efficacy and safety of two Ayurvedic dosage forms for allergic rhinitis: Study protocol for an open-label randomized controlled trial” described in https://doi.org/10.1186/s13063-019-4004-1 [see https://trialsjournal.biomedcentral.com/articles/10.1186/s13063-019-4004-1 for more details]. Diagnosis of knee osteoarthritis will be made according to history, clinical examination findings, and classical radiological findings (ACR functional class I, II, or III) and fulfilling the American College of Rheumatology (ACR) classification criteria, radiographic evidence of OA will be based on the ranking score of the Kellgren-Lawrence radiographic system, and pain severity will be based on VAS. Among them, the participants meeting the inclusion and exclusion criteria will be recruited for the study. All baseline assessment forms (WOMAC, KOOS, and VAS) will be completed by the investigators. Patients will not be allowed to take any other medicines during the trial period. If they have to take any other medicine, they should inform the investigators and discontinue the trial.

#### Baseline assessment

WOMAC, KOOS, and VAS and hematological and biochemical investigations (FBS, FBC, ESR, CRP, ALT/AST, serum creatinine, EGFR, UFR, lipid profile) will be assessed at baseline.

#### Randomization and sequence generation

Randomization for the parallel treatment arms will be carried out by the principal investigator checking the inclusion and exclusion criteria. The randomization sequence will be generated is the same process as described in the protocol for the trial “Efficacy and safety of two Ayurvedic dosage forms for allergic rhinitis: Study protocol for an open-label randomized controlled trial” described in https://doi.org/10.1186/s13063-019-4004-1 [see https://trialsjournal.biomedcentral.com/articles/10.1186/s13063-019-4004-1 for more details]. Block randomization will be done using blocks of 10 to generate the randomization schedule for 140 patients. The patients will be allocated to treatments based on the randomization sequence generated. Two week’s supplies of the assigned investigational products will be handed over to the patients according to the randomized allocation. Each group will be enrolled with an allocation ratio 1:1.

#### Concealment mechanism

The allocation for each randomization number will be put into individually seal opaque envelopes. The envelopes and allocation sequence will be kept under lock and key by one investigator not involved in recruiting patients. The patients meeting the inclusion–exclusion criteria and recruited into the study will be assigned a randomization number sequentially, according to the date and time of recruitment. The allocated treatments indicated in the sealed envelope for each randomization number will be supplied to each patient (i.e., those randomizing do not have access to future allocations). There are no stratification factors are used. To monitor compliance and adverse events, two weekly follow-ups will be done at the Borella Ayurveda Teaching Hospital.

**Fig. 1:**
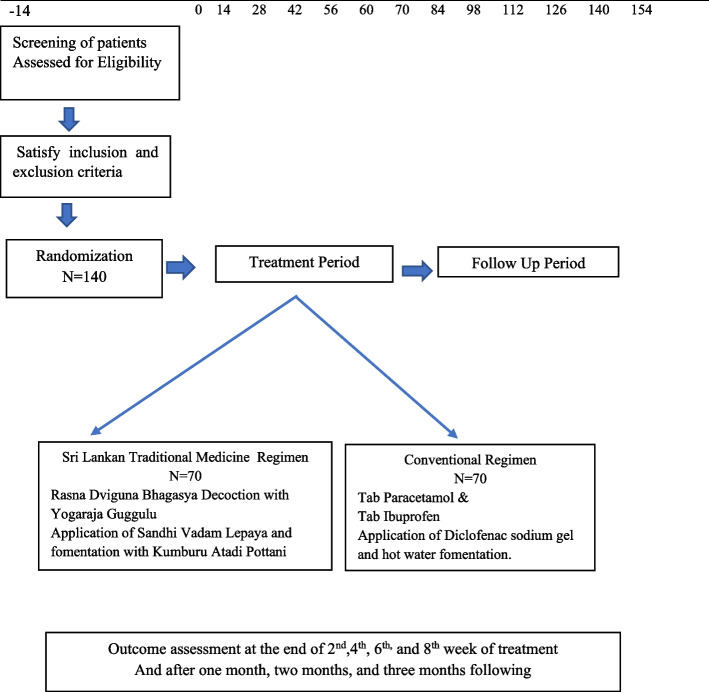
Flow chart of study design

Participants will be assigned to the open-labeled study and provided written informed consent before taking part for 8 weeks of regimen 1 (arm I) or regimen 2 (arm II). Outcomes will be assessed by weekly follow-up at the Borella National Ayurveda Teaching Hospital, and each assessment detail of the outcome study will be informed to the participant. The study design flow chart is shown in Fig. 1.

## Intervention

### Investigational products

#### Sri Lankan traditional medicine regimen

Product I—Rasnadvigunabhagasaya decoction (RDBD) and Yogaraja Guggulu.

Traditional RDBD is a brown-colored liquid, prepared using 80 g *Alpinia calcarata* and 1.6 g other 24 plant ingredients: *Tragia involucrate*, *Sida cordifolia*, *Ricinus Cummunis*, *Cedrus deodara*, *Curcuma zedoaria*, *Crocosmia aurea*, *Justicia adhatoda*, *Zingiber officinale*, *Terminalia chebula*, *Piper chavya*, *Cyperus rotundus*, *Boerhavia diffusa*, *Tinospora cordifolia*, *Argyreia populifolia*, *Anethum sowa*, *Tribulus terrestri*, *Withania somnifera*, *Aconitum heterophyllum*, *Cassia fistula*, *Asparagus racemosus*, *Piper longum*, *Nilgirianthus ciliates*, *Coriandrum sativum*, and *Solanum indicum.* The dried plant materials of the 25 ingredients will be ground separately to pre-prepare a coarse powered pack weighing 120 g containing 25 ingredients; 120 g of dried powder pack of decoction will be used to prepare the decoction needed for 2 days. A 2-week supply (seven packs) of these pre-prepared dried herbs pack will be supplied to the patient. They will be informed to put the supplied herbal pack into an earthen pot or stainless-steel pot, add 3840 ml of water, and simmer under low flame until the volume is reduced to 480 ml. The process of preparation under standard conditions will be demonstrated to the patients who are selected for the RDBD arm at the Department Dravyaguna Vignana or Department of Kayachikithsa of Institute of Indigenous Medicine using video. They will be requested to take a daily dose of 120 ml of decoction before breakfast (6 am) and before dinner (6 pm) daily rest for the next day before breakfast (6 am) and before dinner. Pills of Yogaraja Guggulu will be purchased from Sri Lanka Ayurveda Drug cooperation, and 56 pills will be packed in light and waterproof polythene bags and provided to take along with the decoction for rescue pain.

#### Product II—Sandhi Vadam Lepaya (SVL)

Pastes or Lepa or Paththu will be prepared according to the standardized and quality-controlled methods described in Sri Lankan traditional medicine. Paththu will be given for the external application on the knee prepared by 15 ingredients: *Asparagus racemosus* (both tube and leaves) 50 g, *Crataeva nurvala* (leaves and bark) 50 g, *Moringa oleifera* (bark) 50 g, *Vitex negundo* (leaves and bark) 50 g, *Caesalpinia bonduc* (leaves and bark) 50 g, and *Alstonia scholaris* (bark) 50 g pounded well, and take 1920 ml juice after squeezing and heating up to 480 ml. *Piper nigrum* (fruit) 10 g, *Allium sativum* (bulb) 5 g, *Zingiber officinale* (rhizome) 5 g, *Pipper longum* (fruit) 5 g, *Ferula asafoetida* (resin) 5 g, *Trachyspermum ammi* (seeds) 5 g, *Cuminum* cyminum (seeds) 5 g, and *Caesalpinia bonduc* (seeds) 5 g grounded separately and grounded with *lime juice*, and add it to 240 ml of lime juice and mix well. Add this mixture to the previously prepared juice, and keep it in flame until it turns thick by evaporating its water content. One packet of 50 g in light and waterproof packed will be provided to use for 1 day. Fourteen packets will be provided, evenly applied over the knee; it should be covered with a cotton wool and properly bandaged with a gauze bandage. Keep it for 8 h and removed, and ask to cleanse the knee area properly.

#### Product III—Kumburuatadi Pottaniya (KAP)

Herbal fomentation bollus (Pottaniya) was prepared with 10 ingredients: *Caesalpinia bonduc* (seed pulp) 7.5 g, *Ferula asafoetida* (resin) 7.5 g, *Zingiber officinale* (rhizome) 7.5 g, *Pipper longum* (fruit) 7.5 g, *Allium sativum* (bulb) 7.5 g, *Terminalia chebula* (fruit) 7.5 g, *Maduka Indica* (seed) 7.5 g, *Ricinus communis* (seed) 7.5 g, *Sesamum indicum* (seed) 7.5 g, and *Cococus nucifera* (seed pulp) 7.5 g taken and pounded well, and make a pollute and steam it. Dip it in a heated mixture of oils of Mee thel (oil extracted from seeds of *Maduka Indica*), Erdu thel (oil extracted from seeds of *Ricinus communis*), Thala thel (oil extracted from seeds of *Sesamum indicum*), Elagithel (Ghee), and Polthel (oil extracted from seed pulp of *Cococus* nucifera) collectively known as Pasthel (five types of oils) used commonly in Sri Lankan traditional medicine. Two pottani will be given to the patient, and they will be informed to steamed properly and dip in lightly heated mixture of 5 ml oil from each abovementioned five oils (Mee, Eradu, Thala, Ethagithel, and Pol thel), and fomentation was done for 30 min after removing the paste.

Product IV—tablet paracetamol and tablet ibuprofen.

Individualized conventional care for knee OA according to current practices, non-steroidal anti-inflammatory drugs (NSAIDs)—paracetamol (500 mg) and ibuprofen (200 mg)—as rescue medication were selected as the comparator for this clinical trial. The total quantity of paracetamol and ibuprofen required for the clinical trial, from one of the leading brands of paracetamol, and ibuprofen will be purchased from one single batch, directly from the State Pharmaceutical Company (SPC) for the purpose of the trial. The purchased products will be stored at 25 °C in an air-conditioned environment at the IIM. Patients allocated to the conventional arm are requested to take a daily dose of two tablets of paracetamol (500 mg) and two tablets of ibuprofen (200 mg) twice a day after breakfast and after dinner.

Product V—diclofenac sodium gel and fomentation with warm water.

Topically applied NSAIDs reduce systemic exposure compared with oral NSAIDs, and European guidelines recommend their use. The NSAID is available in a range of topical formulations. The topical formulation of 2% diclofenac sodium gel aims to reduce adverse systemic problems, and fomentation with warm water is recommended before applying it. The total quantity of 2% diclofenac sodium gel required for the clinical trial, from one of the leading brands of 2% diclofenac sodium gel, will be purchased from one single batch, directly from the State Pharmaceutical Company (SPC) for the purpose of the trial. The purchased products will be stored under 25 °C in an air-conditioned environment at the IIM. Patients allocated to the conventional arm are requested to apply 2% diclofenac sodium gel over the knee and carried fomentation with warm water once a day.

Details of the investigational drugs are shown in Table 1. Improving patient compliance is the same process as described in the protocol for the trial “Efficacy and safety of two Ayurvedic dosage forms for allergic rhinitis: Study protocol for an open-label randomized controlled trial” described in https://doi.org/10.1186/s13063-019-4004-1 [see https://trialsjournal.biomedcentral.com/articles/10.1186/s13063-019-4004-1 for more details].

**Table 1:** Investigational products

Treatment regimen	Treatment duration	Interventional group—arm I	Conventional group—arm II
External thermotherapy	30 days	Fomentation (pottani)—KAP 100 g daily	Hot water Sufficient amount daily
Topical application	30 days	Paste—SVL Necessary amount daily	2% diclofenac sodium gel Sufficient amount daily
Internal	60 days oral	Rasnadvigunabhagasaya decoction—RDBD 120 ml × bd before meals	Tab paracetamol (500 mg × 2) × bd After meals
Rescue medication	60 days oral	Yogaraja Guggulu pill (500 g × 2) × bd before meals	Tablet ibuprofen (200 mg × 2) × bd After meals
Follow-up after every 2 weeks for 3 months (no intervention)			

#### Storage, packaging, and dispensing of investigational drugs

Decoction, paste, and pottani will be manufactured and other drugs will be purchased from the Sri Lanka Ayurvedic Drug cooperation. Decoction, paste, and pottani drugs used for the study will be prepared at the pharmacology department of IIM under GMP conditions. Decoction drugs, pottani, pills, and tablets will be packed in light and waterproof polythene, and pottani will be packed in light and waterproof sealed plastic container. Each drug will be packed for 14 days and labeled which indicates the cord number, arm, dose, time of administration, and mode of administration (oral or external use, etc.). These will be stored in the clinic of the Ayurveda Teaching Hospital, Sri Lanka, to be provided to randomized patients according to the predetermined allocation sequence. A supply of drugs for 14 days will be dispensed to the study participants at two weekly visits with instructions and patient diaries will be provided for all.

### Outcome measurements

#### Primary outcome

The primary endpoint is a score on the Western Ontario and McMaster University Osteoarthritis Index (WOMAC) measured at the baseline and the end of the intervention (after 8 weeks).

#### Secondary outcome

Secondary outcome measurements will be the change in Knee Injury and Osteoarthritis Outcome Score (KOOS), a pain disability index, a visual analog scale for pain and sleep quality, and a quality-of-life index (The World Health Organization (WHO), the WHOQOF: https://www.who.int/tools/whoqol) measured at the baseline and the end of the intervention (after 8 weeks and 1 month, 2 months, and 3 months of follow-up).

Changes in ESR and CRP count will be studied by comparing before and after treatment values. Procedures related to the study are shown in Table 2.

**Table 2:** Study procedures

Study period													
	Screening Run in period	Recruitment	Treatment								Follow-up		
**Time point (weeks)**	− 1	0	1	2	3	4	5	6	7	8	12	16	20
Eligibility Screening	+												
Informed consent	+												
Recruitment		+											
Randomization			+										
**Arms**													
Arm I—STM													
Arm II Conventional													
Investigations	+		+							+	+	+	+
**Assessments**													
WOMAC	+		+							+	+	+	+
KOOS	+		+							+	+	+	+
Pain disability index	+		+							+	+	+	+
Visual analog scale for pain	+		+							+	+	+	+
Sleep quality	+		+							+	+	+	+
Quality-of-life index	+		+							+	+	+	+
Adverse events			+	+	+	+	+	+	+				

#### Safety assessment

The same process as described in the protocol for the trial “Efficacy and safety of two Ayurvedic dosage forms for allergic rhinitis: Study protocol for an open-label randomized controlled trial” described in https://doi.org/10.1186/s13063-019-4004-1 [see https://trialsjournal.biomedcentral.com/articles/10.1186/s13063-019-4004-1 for more details].

#### Data handling, record keeping, and dissemination

The same process as described in the protocol for the trial “Efficacy and safety of two Ayurvedic dosage forms for allergic rhinitis: Study protocol for an open-label randomized controlled trial” described in https://doi.org/10.1186/s13063-019-4004-1 [see https://trialsjournal.biomedcentral.com/articles/10.1186/s13063-019-4004-1 for more details].

#### Ethical considerations

The approval of the research protocol has been obtained from the research approval committee of the Faculty of Graduate Studies, University of Colombo, and the Ethics Review Committee of the Institute of Indigenous Medicine, University of Colombo. The trial was registered in ISRCTN registry (Trial Number ISRCTN58050062 https://doi.org/10.1186/ISRCTN58050062). The study will be conducted adhering to Good Clinical Practice (GCP) guidelines and ethical consideration is the same process as described in the protocol for the trial “Efficacy and safety of two Ayurvedic dosage forms for allergic rhinitis: Study protocol for an open-label randomized controlled trial” described in https://doi.org/10.1186/s13063-019-4004-1 [see https://trialsjournal.biomedcentral.com/articles/10.1186/s13063-019-4004-1 for more details].

#### Protocol amendments

Protocol modifications will be informed to ethics review committee and the trial registry approval. Participants who enrolled before the amendments also will notify of the changes done.

## Method of data analysis

In this study, data analysis will be performed by using the SPSS version 21.0 (SPSS Inc., Chicago, Illinois) software. The level of significance will be established at *α* = 0.05. Missing data at outcome assessment will be replaced with the available latest values of the outcome measure. Intention-to-treat analysis will be performed for all efficacy outcomes and safety outcomes. In addition, a per-protocol analysis will be performed for the efficacy outcomes by including only the patients completing the follow–up. Participants who do not adhere to the interventions described in the protocol will be excluded. Descriptive statistics will be used to describe baseline characteristics in the two study arms. Both study arms will be described and compared at baseline using a table including relevant baseline variables. Two-sided statistical tests will always be performed.

The primary outcome is a change in the score on the WOMAC Index after 8 weeks presented as WOMAC means per group with 95% confidence intervals and a two-sided *P* value for the treatment group and comparison. A paired *t*-test will be used to compare the mean of quantitative variables with normal distribution in each group between the beginning and the end of the study, and the *t*-test will be used to compare the mean between the two groups at the beginning and end of the study. In the case of quantitative variables with non-normal distribution, Wilcoxon and Mann–Whitney tests are used, respectively. In the case of the variables measured three times during the study (beginning, fourth week, and eighth week), a repeated ANOVA test will be used. Covariance analysis will be used to eliminate the effect of quantitative confounding factors. Quantitative confounding factors are physical activity and baseline values of the biochemical markers. A chi-square test will be used to compare the qualitative variables between the two groups and regression analysis is used to eliminate the effect of qualitative confounding.

## Discussion

Knee OA is a chronic joint disease with significant health and psychological burden to patients due to persistent pain for prolonged period. Recent guidelines have addressed the many treatments that aim to relieve symptoms, in particular pain and improved function and not provide a cure for OA. There are numerous drug treatments for osteoarthritis; however, their efficacy and adverse effect profiles often limit their use. The drug approval process of structure-modifying drugs allowing the use of surrogate markers which may be reasonably likely to predict important clinical outcomes would increase the potential for the development of therapies of OA where currently there is no known cure and no interventions available to stop the progression or manage the symptoms with an acceptable benefit to harm profile [7]. Therefore, effective treatment would be important for treating this disease. Due to fear of side effects and adverse effects of these allopathic treatment, presently, most of their interest focused towards CAM and in Sri Lanka; also, many of the people are seeking treatment regimens for knee OA from Sri Lankan traditional medicine. Rasandivgunabhagasya decoction is an herbal decoction that has been prescribed for knee OA with Yogaraja Guggulu as an internal medicine, and Kumburuatadi pottaniya and Sandivadam lepaya were used for external local application in Sri Lankan traditional medicine as a treatment regimen for a long time. Our research team designed these two arms, an open-labeled parallel group randomized control trial to compare and evaluate the effectiveness of this regimen in knee OA compared to pain management done with a nonsteroidal anti-inflammatory drug treatment regimen. This clinical trial will be able to provide evidence-based scientific data on the Sri Lankan traditional medicine regimen in the treatment of knee OA. This trial is also expected to develop the capacity to scientifically evaluate Sri Lankan traditional medicine treatment regimens that could help patients having chronic joint disorders such as knee OA.

## Strengths and limitations

To our knowledge, this is the first randomized controlled clinical trial to investigate the efficacy of the Sri Lankan traditional medicine regimen in Sri Lanka for knee OA. The results of this study will provide evidence regarding the use of this Sri Lankan traditional medicine regimen for treatment in knee OA. Any drugs containing heavy metals as ingredients cannot be used directly for human trials due to ethical issues. Therefore, only the herbal plant-based drug formulae were selected for the study. The participants are not allowed to use any other medication during the trial period and have to discontinue the trial if they do. This may lead to informative drop-out.

## Trial status

The recruitment is currently in progress and it is expected that the recruitment will be completed by the end of August 2022.

## Data Availability

After the study is completed, the data will be made available on request from the corresponding author.

## References

[1] American College of Rheumatology (ACR) Guideline for the Pharmacologic and Non-Pharmacologic Management of Osteoarthritis of the Hand, Hip and Knee *Project Plan – September 2017*[Internet] Available from https://www.rheymatology.org/Portals/0/Files/OA-Guideline- Project Plan.pdf. [Accessed 06^th^ February 2019].

[2] World Health Organization. Essential medicines and health products, Priority diseases and reasons for inclusion [Internet]. Available from https://www.who.int/medicines/areas/priority_medicines/prior_med_ch6_12/en/ [Accessed 06^th^ February 2019].

[3] Osteoarthritis: A Serious Disease, Submitted to the U.S. Food and Drug Administration. December 1, 2016 . Available from https://www.oarsi.org/.../2016/oarsi_white_paper_oa_serious_disease_ 121416_1.pdf. [Accessed 27th December 2018].

[4] Mat S, Jaafar MH, Ng CT, Sockalingam S, Raja J, Kamaruzzaman SB (2019). Ethnic differences in the prevalence, socioeconomic and health related risk factors of knee pain and osteoarthritis symptoms in older Malaysians. PLoS ONE.

[5] Pal CP, Singh P, Chaturvedi S, Pruthi KK, Vij A (2016). Epidemiology of knee osteoarthritis in India and related factors. Indian J Orthop.

[6] Osteoarthritis: Serious disease, Submitted to the U.S. Food and Drug Administration December 1, 2016 Available from https://www.oarsi.org/sites/default/files/docs/2016/oarsi_white_paper_oa_serious_disease_12 1416_1.pdf

[7] Gnanavimala K (1963). Yogarnavaya.

[8] Gnanavimala K (1948). Proyoga Rathnavali.

[9] Vilegoda D (1967). Bhesajja Manjusa Nuthana Sannasaya.

[10] Kodikara SS (1909). Kashaya Sagaraya.

[11] Godamune SD (1992). Kashaya Sangraha.

[12] Department of Ayurveda (1992). Thalpathe Pilium.

[13] Kumarasinghe A (1992). King Buddhadasa’s Sarartha Samgrahaya.

[14] Kumarasinghe A (1987). Vaidyaka Sarasamkshepaya of Rajaguru Sri Chandra.

[15] Kanjiv L (2008). Bhaisajya Ratnavali of Shri Govinda Dasji.

[16] Dash V, Sharma RK (2009). Caraka Samhita: Text with English Translation and Critical Exposition based on Cakrapani Datta’s Ayurveda Dipika.

[17] Sharma PV. Susruta Samhita. Varanasi: Chaukhamba Orientalia Publications.; 2005.

[18] Murthy KRSK (2005). Astanga Samgraha of Vagbhata.

[19] Murthy KRSK. Bhava Prakasha of Bhavamishra. Varanasi: Chaukhamba Krishnadas Academy Publications; 2009.

[20] Murthy KRSK (2009). Madhava Nidana of Mahavakara.

[21] Murthy KRSK. Sarngadhara Samhita. Varanasi: Chaukambha Orientala Publications; 2006.

[22] Kessler CS, Dhiman KS, Kumar A, Ostermann T, Gupta S, Morandi A (2018). Effectiveness of an Ayurveda treatment approach in knee osteoarthritis a randomized controlled trial. Osteoarthr Cartil.

